# Molecular choreography of vesicle trafficking: Exocyst–SNARE interplay in plant defense

**DOI:** 10.1016/j.jare.2026.01.059

**Published:** 2026-01-26

**Authors:** Muhammad Adnan, Liu Xiaokun

**Affiliations:** Jiangxi Provincial Key Lab of Plant Germplasm Resources Innovation and Genetic Improvement, Lushan Botanical Garden, Chinese Academy of Sciences, Jiangxi, China

**Keywords:** Vesicle trafficking, Exocyst complex, SNARE proteins, Defense related secretion, Plantimmunity

## Abstract

**Background:**

The plant secretory system operates as a precision logistics network that sustains cellular homeostasis and mobilizes rapid defense against pathogens. Central to this system are SNARE (soluble *N*-ethylmaleimide-sensitive factor attachment protein receptor) proteins, which catalyze membrane fusion, and the octameric exocyst complex, which tethers *trans*-Golgi–derived vesicles to defined plasma-membrane domains. Rather than functioning independently, these machineries act as an interdependent module: the exocyst facilitates vesicle tethering and promotes SNARE complex assembly, while SNARE proteins reciprocally stabilize exocyst positioning and activity. This synergy ensures the targeted delivery of immune cargos—including pathogenesis-related proteins, callose synthases, transporters, and specialized metabolites—directly to pathogen attack sites. However, pathogens deploy effectors that disrupt exocyst–SNARE interfaces, weakening these defensive supply lines.

**Aim of review:**

This review integrates recent advances that expand the functional repertoire of both complexes across canonical and unconventional secretion pathways. It incorporates regulatory factors such as Rab GTPases, Rho-GTPases of plants (ROPs), phosphoinositides, cytoskeletal elements, and post-translational modifications, offering a comprehensive framework for understanding exocyst–SNARE coordination in plant defense. At the same time, it highlights key unresolved questions, such as the molecular choreography of holo-exocyst–SNARE assemblies in vivo, rules of immune cargo selection, and the dynamic rerouting of pathways during stress.

**Key scientific concepts of review:**

A central theme emerging from these insights is the cooperative interaction between exocyst and SNARE proteins as the basis for localized immune delivery. However, the vulnerability of this machinery to pathogen interference underscores the need for deeper mechanistic understanding. This review positions vesicle-fusion control as a promising and tractable strategy for engineering durable disease resistance in crops, framing exocyst–SNARE biology as a critical lever in strengthening plant immunity.

## Highlights

• Proposes a unified tether–handover–fusion model linking exocyst capture to SNARE-driven membrane fusion at immune foci.
• Shows how EXO70 isoforms scaffold and position immune SNARE modules (SYP121/132–SNAP33–VAMP721/722) to deliver PR proteins and callose.
• Integrates regulatory cues—MAPKs, Ca^2+^/SYT5, RabA/RabE–Sec15, and lipids—to explain when and where defense secretion occurs.
• Synthesizes pathogen strategies that sabotage this node (e.g., XopP–EXO70B, AVR-Pii–Exo70F2/3, HopM1–MIN7) and their phenotypic outcomes.
• Identifies actionable gaps (cargo routing, Rab–exocyst–SNARE triads, EV linkage) and outlines tractable experiments to close them.

## Graphical abstract

**Figure:**
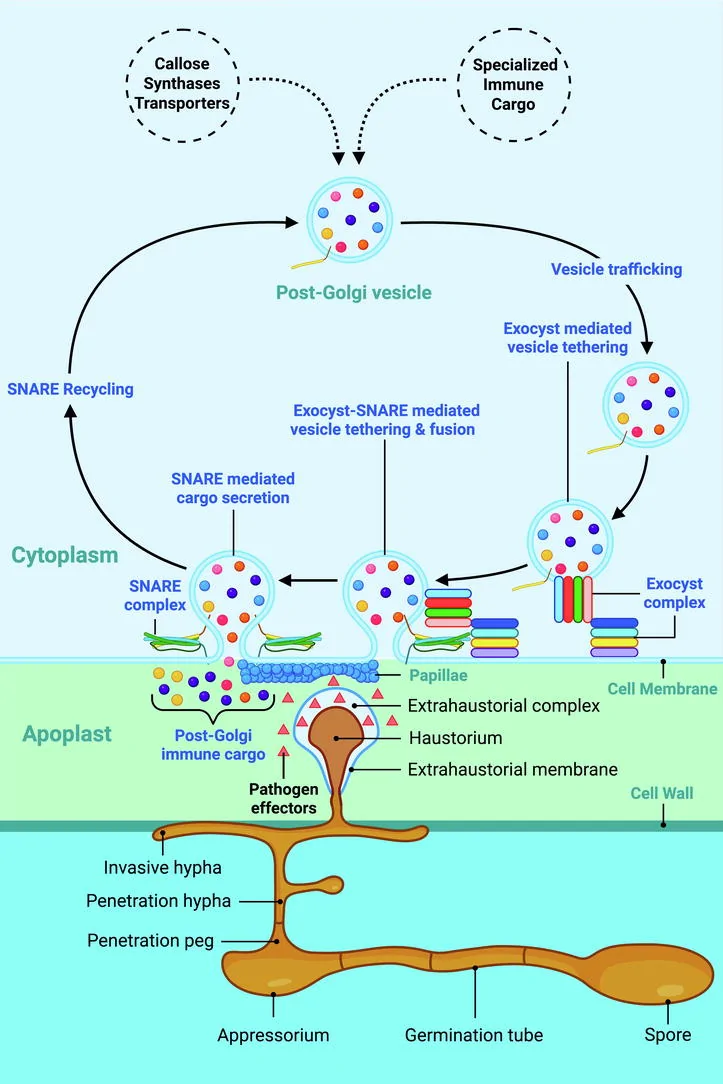

## Introduction

Plants survive pathogen attack by coupling rapid immune perception with precisely targeted secretion [1]. At the plasma membrane (PM), recognition of invading microbes triggers delivery of defense cargo—pathogenesis-related (PR) proteins, cell wall–reinforcing enzymes, and antimicrobial metabolites—into the apoplast and cell wall [2]. This response relies on two core machineries: the exocyst tethering complex, which positions secretory vesicles at defined cortical sites, and the soluble *N*-ethylmaleimide-sensitive factor attachment protein receptor (SNARE) system, which drives membrane fusion and cargo release [3], [4], [5].Fig. 1.Table 1.Table 2..

**Fig. 1:**
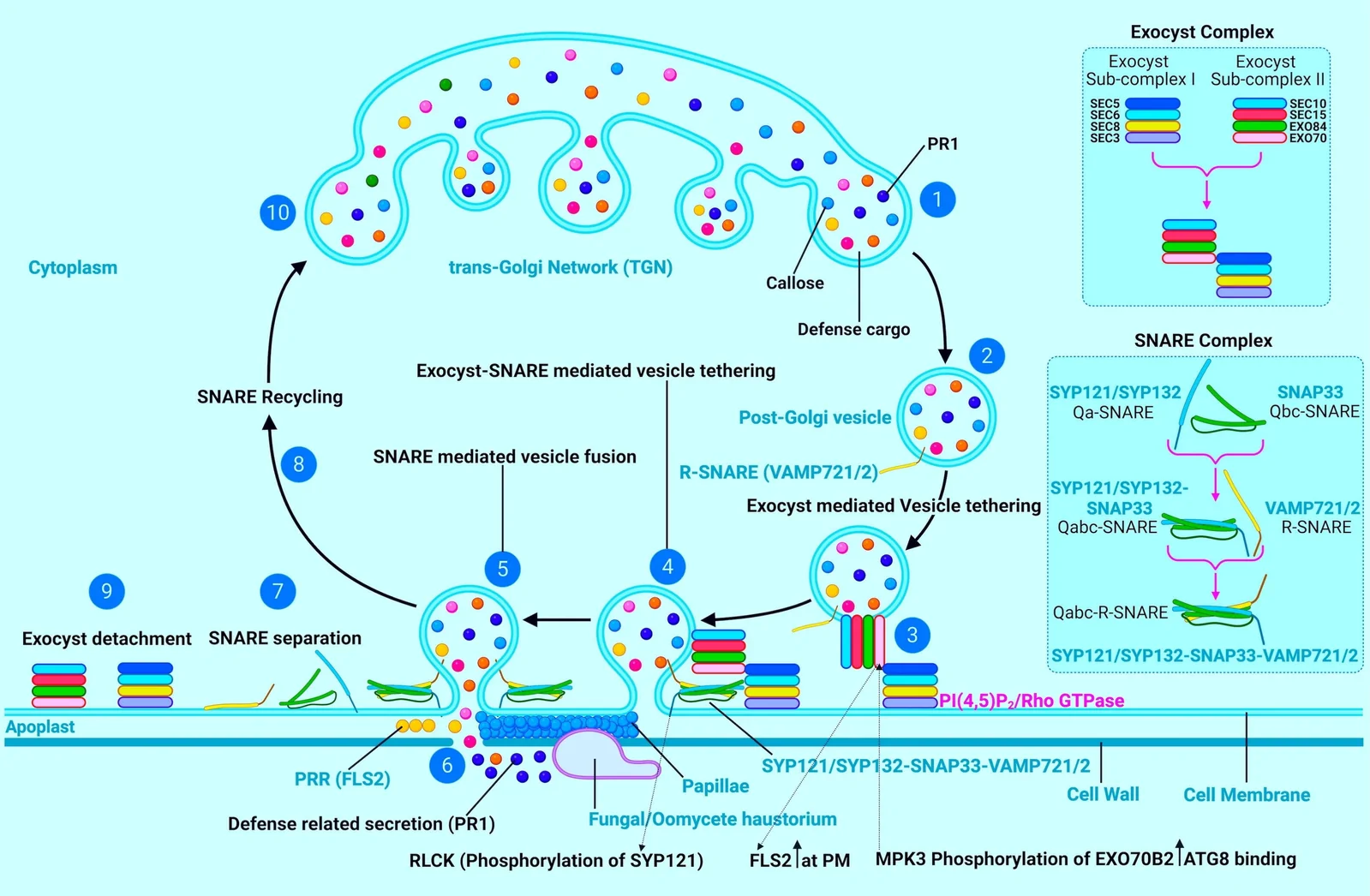
TGN-to-plasma-membrane defense secretion: exocyst–SNARE choreography targets immune cargo to pathogen interfaces. Schematic of the plant exocytic route that delivers defense cargo from the TGN to defined PM nanodomains during immune activation. The exocyst tether and the SYP121/SYP132–SNAP33–VAMP721/722 SNARE module act sequentially to ensure spatially precise secretion at pathogen entry sites (papilla/encasement). **Process steps (blue circles): 1. Cargo sorting at the TGN.** PR1, callose synthase/precursors, and other defense proteins are packaged into post-Golgi carriers. The vesicle membrane incorporates the R-SNARE VAMP721/722. **2. Vesicle budding and export.** Coated carriers leave the TGN loaded with immune cargo; VAMP721/722 marks them for PM fusion. **3. Targeting to PM nanodomains.** Exocyst subunits (especially EXO70) are recruited to PI(4,5)P_2_- and ROP (Rho of plants) GTPase-enriched PM regions near PRRs e.g., flagellin-sensing 2 (FLS2). Subcomplex I (SEC3/5/6/8) and subcomplex II (SEC10/15/EXO70/EXO84) assemble into the holocomplex at these sites. **4. Vesicle tethering/docking.** The exocyst captures incoming VAMP721/722-positive vesicles and positions them over the target nanodomain; EXO70 can engage the longin domain of VAMP721/722. In parallel, the binary PM Q-SNARE complex SYP121–SNAP33 forms. **5. SNARE-mediated membrane fusion.** VAMP721/722 (R) pairs with SYP121/SYP132 (Qa) and SNAP33 (Qbc) to form the *trans*-SNARE four-helix bundle, driving bilayer merger and pore opening. **6. Focused defense secretion at infection sites.** Cargo is released to the apoplast at papilla/haustorial interfaces; callose and PR proteins accumulate. PRR signaling e.g., receptor-like cytoplasmic kinase (RLCK)-dependent phosphorylation of SYP121 reinforces local secretion. MPK3-dependent phosphorylation of EXO70B2 later recruits ATG8 for negative feedback. **7. SNARE complex disassembly.** α-SNAP/Sec17 and NSF/SEC18 (not shown) use ATP to separate *cis*-SNAREs after fusion. **8. SNARE recycling.** Disassembled SNAREs are retrieved: VAMP721/722 is endocytosed and returned to the TGN; PM Qa/Qbc SNAREs recycle locally for subsequent rounds. **9. Exocyst detachment and turnover.** After fusion, the exocyst disengages; phosphorylated EXO70B2 can bind ATG8 and be cleared by selective autophagy, resetting tether availability. **10. Reloading at the TGN.** Recycled SNAREs and lipids re-enter the TGN to support continued rounds of immune secretion. **Insets (right)** depict the two exocyst sub-complexes and the composition of the ternary SNARE bundle. **Exocyst complex.** Two tetrameric sub-assemblies—Subcomplex I (SEC3, SEC5, SEC6, SEC8) and Subcomplex II (SEC10, SEC15, EXO70, EXO84)—associate to form the octameric tether. **SNARE complex.** The ternary fusion module at the PM comprises SYP121/SYP132 (Qa), SNAP33 (Qbc; two helices), and VAMP721/722 (R), which zipper from N- to C-terminus to catalyze fusion.

**Table 1:** SNAREs–exocyst subunits implicated in plant defense.

A-Exocyst subunits implicated in plant defense						
Protein (Gene)	Primary defense role	Cargo/Process most affected	Pathogen/Trigger	Key partners	Evidence	References
EXO70B1	Raises receptor abundance at PM	PRR provisioning and PTI outputs	*P. syringae*	EXO70B2/LRR-RK	Genetics, PM proteomics, microscopy	[16]
EXO70B2	Papilla/encasement formation	Callose/lignin appositions; focal secretion	*Blumeria graminis f. sp. hordei*	SEC6 (exocyst core), SYP121	Genetics, co-localization, direct interaction	[8], [16]
EXO70B2	Immune feedback control of tether pool	Autophagic turnover via ATG8 after MPK3 phosphorylation	Flagellin 22 (flg22)/PTI	MPK3, ATG8	Phospho-mutants, co-IPs, confocal microscopy	[16]
EXO70A1	Positions immune SNAREs at PM	SYP121/SNAP33/VAMP721 localization	General PTI	SYP121, VAMP721	Direct binding; epistasis with VAMP721	[3]
EXO70FX12 (FX clade)	Required for RK immunity	RK signaling throughput; secretory focus	*P. striiformis f.sp. tritici*	Rps8/Pur1 (LRR-RK)	Map-based cloning; complementation	[6]
OsEXO70F2/F3	Effector target; part of NLR-ID surveillance	Effector docking on EXO70 pocket	*Magnaporthe oryzae* AVR-Pii	NLR pair Pii; AVR-Pii effector	Crystal structure of AVR-Pii–EXO70F2; mutagenesis	[83], [92]
EXO70B (family)	PR1 and callose delivery to PM	General immune secretion	*Xanthomonas* spp.	XopP	Effector assays, Y2H, PTI readouts	[93]
EXO70H1	Contributes to bacterial/fungal defense	Callose; defense secretion	*P. syringae*; *B. graminis f. sp. hordei*	EXO70B2, SEC3A	Mutant phenotypes	[84], [88]
Exocyst core (SEC6)	Co-localizes with EXO70B2 at papillae; structural node	Focal membrane domain organization	*B. graminis f. sp. hordei*	EXO70B2, SYP121	Co-localization	[17]
B-SNAREs (and helpers) implicated in plant defense						
SYP121/PEN1–SNAP33–VAMP721/722	Penetration resistance; focal secretion at papillae/encasements	Cell-wall enzymes; defense cargo	*M. oryzae*	EXO70A1, EXO70B2, ARF-GEF	Genetics, imaging, biochem.; epistasis	[19]
SYP132–SNAP33–VAMP721/722	Secretion of PR1/PR2/PR5; basal resistance	PR1 export to apoplast; SA-linked	*P. syringae*	EXO70B1/B2	Secretory proteomics; genetics	[2], [22]
SYT5 (helper)	Facilitates SYP132–VAMPs assembly	Boosts immune exocytosis throughput	*P. syringae*	SYP132, VAMP721/722	Biochem., immunity assays	[2], [22]
SNAP33	Qbc-SNARE in immune fusion hubs	General defense exocytosis	*V. dahliae*	SYP121/132; VAMP721/722	Genetics, proteomics	[14]
VAMP721/722 (R-SNAREs)	Vesicle-side fusogens for PM delivery	Broad immune cargo; growth-essential	Multiple pathogens; PTI	SYP121/132; EXO70A1	Genetics, biochem., imaging	[15], [22], [41], [94]
SYP121/SYP122 (parallel Qa routes)	Parallel PM tracks with distinct cargo biases	Cell-wall proteins vs. others	Bacteria/fungi (context-specific)	SNAP33; VAMP721/722	MS cargo mapping; genetics	[20]
SYP121 (phospho-control)	Phospho-tuning links signaling to exocytosis	Defense related stomatal/PM transport	Abiotic; immune cross-talk	RLCK	Phospho-mutants	[20]

**Table 2:** Pathogen effectors targeting the plant exocyst–SNARE vesicle trafficking machinery.

Pathogen /Effector	Effector Category	Targeted component (molecular target)	Mechanism of Action	Consequence for Plant Immunity	References
*P. syringae* AvrPtoB	Type III effector	Exocyst (EXO70B1)	E3 ubiquitin ligase-mediated ubiquitination, proteasomal degradation	Disruption of vesicle tethering; reduced secretion of defense molecules	[113]
*P. syringae* AvrRpt2	Type III effector	Exocyst–regulator link (RIN4/EXO70 link)	Cysteine protease cleavage of RIN4, cytoplasmic release of EXO70B1	Loss of PM-localized EXO70B1; impaired papilla formation	[115], [116]
*P. syringae* AvrRpm1	Type III effector	Exocyst–regulator link (RIN4/EXO70 link)	ADP-ribosylation of RIN4	Altered RIN4 function; reduced defense signaling	[117], [118]
*Ralstonia solanacearum* RipE1	Type III effector	Exocyst (EXO70B1)	Proteasomal degradation	Compromised resistance; reduced vesicle targeting to PM	[119]
*Phytophthora infestans* AVR1	RXLR effector	Exocyst (SEC5)	Direct binding; inhibits vesicle tethering	Suppressed callose deposition at infection sites	[114]
*P. infestans* PexRD12/31	RXLR effector	SNARE (VAMP72x)	Interference with vesicle fusion machinery	Reduced delivery of PR proteins	[25]
*X. campestris* XopP	Type III effector	Exocyst (SEC10, EXO70, EXO84)	Blocks exocyst holocomplex assembly	Impaired PM tethering; secretion failure	[93]
*M. oryzae* AVR-Pii	AVR effector	Exocyst (OsEXO70F2/F3)	Direct association; immune recognition via NLR	ETI activation; resistance	[92]
*Phytophthora sojae* PsAvh181	RxLR effector	SNARE (SNAP-1)	Disrupts SNAP-1–NSF interaction	Blocks PR1 secretion	[26]
*Hyaloperonospora arabidopsidis*/HaRxL17 and HaRxL77	RXLR effectors	RabA GTPases	GTPase (Inhibits vesicle targeting)	Reduced secretion at infection sites	[120], [121]

Over the past decade, plant endomembrane research has progressed from cataloging trafficking components to elucidating how secretion is spatially reprogrammed during immunity. Work in Arabidopsis (*Arabidopsis thaliana*) and cereals now supports a coordinated exocyst–SNARE relay in which the exocyst captures post-Golgi vesicles at phosphoinositide-enriched PM nanodomains, followed by SNARE-mediated fusion that enables rapid, focal delivery of defense cargo at infection sites [3], [4], [5], [6], [7], [8].

Mechanistically, plant SNAREs assemble into a 3Q:1R four-helix bundle: PM Q-SNAREs syntaxin of plants 121 (SYP121) or SYP132 pair with the Qb/Qc component synaptosome-associated protein 33 (SNAP33) and engage the vesicular R-SNARE vesicle-associated membrane protein 721/722 (VAMP721/722) to execute fusion at highly restricted cortical domains [9], [10], [11], [12], [13], [14], [15]. This defines a trafficking logic in which exocyst-guided tethering and SNARE-driven fusion act sequentially to achieve subcellular precision in immune secretion.

Two principles follow from this model. First, immune competence is trafficking-dependent: secretory and endomembrane routes regulate the abundance and maintenance of pattern-recognition receptors (PRRs) at the PM, thereby tuning the gain of pattern-triggered immunity (PTI). Second, once signaling begins, exocyst-to-SNARE handover spatially confines fusion to pathogen contact sites, enabling focused secretion of antimicrobial cargo [2], [3], [5], [6], [16], [17]. Genetic and cell-biological evidence supports this hierarchy: exocyst complex subunit 70A1 (EXO70A1) can interact with SYP121 and VAMP721, and the *exo70a1 vamp721* double mutants display severe developmental and immune defects, consistent with an essential tether-to-SNARE relay dominated by the R-SNARE [3], [18]. In parallel, SYP121- and SYP132-centered pathways may operate concurrently yet converge on common VAMP72-type R-SNAREs to drive defense secretion [13], [15], [19], [20], [21], [22].

This relay is dynamically tuned to balance secretory output with cellular homeostasis. Evidence links SYP132 accumulation at the PM to PR1 secretion during bacterial infection, suggesting that SYP132 may function as a cargo-selective node for apoplastic immunity [13], [22], [23]. Conversely, negative feedback can restrain secretion: mitogen-activated protein kinase 3 (MPK3)-dependent phosphorylation of EXO70B2 enhances its association with autophagy-related protein 8 (ATG8), targeting EXO70B2 for autophagic degradation and limiting tether availability once demand subsides [24].

Because this node couples receptor provisioning with antimicrobial export, it represents a high-value target for pathogens. Disrupting exocyst–SNARE coordination can weaken both immune perception and downstream secretion, helping explain why trafficking hubs are repeatedly targeted by microbial effectors [25], [26], [27]. Consistent with this pressure, evolutionary analyses reveal extensive diversification of the EXO70 family—especially the grass-specific EXO70FX clade—likely shaped by recurrent host–pathogen interactions [6], [28], [29]. In grasses, polyploidy may further promote retention and specialization of duplicated EXO70 copies, enabling lineage-specific immune wiring and offering opportunities for crop improvement through selective deployment of EXO70 isoforms.

Despite this progress, key questions remain: how many vesicles are tethered per fusion event; what determines vesicle dwell time and route choice between SYP121- and SYP132-centered pathways; and how do lipid composition, calcium signaling, and extracellular vesicle (EV) pathways intersect with immune secretion? Emerging tools make these problems tractable, including live-cell and super-resolution imaging of PM nanodomains, proximity labeling to map transient tether–SNARE–Rab–kinase neighborhoods, clustered regularly interspaced short palindromic repeats (CRISPR) editing to test binding interfaces and phosphorylation sites in vivo, and quantitative (phospho)proteomics to resolve cargo flux and tether turnover over time [30], [31], [32], [33].

Against this backdrop, our review synthesizes current understanding of how the exocyst and SNARE machineries cooperate to orchestrate plant immunity, outlines the regulatory logic that tunes their interplay, and examines pathogen strategies that exploit this interface. We conclude by highlighting outstanding knowledge gaps—particularly nanodomain organization and feedback control of secretory throughput—and propose a roadmap linking mechanistic trafficking insights to field performance for engineering durable, low-penalty secretory immunity in crops.

## The vesicle trafficking machinery

Vesicle transport underpins most secretory decisions in plant cells—from routine cell-wall remodeling to the rapid export of immune cargo [1], [2]. Although the itinerary is canonical (endoplasmic reticulum [ER] synthesis, Golgi maturation, *trans*-Golgi network [TGN] sorting, and delivery to the PM), execution is highly regulated by coat/adaptor selection, cytoskeleton-based transport, capture at PM nanodomain, SNARE assembly, and membrane fusion [5], [34], [35]. Recent work in cereals and Arabidopsis has clarified how the exocyst (a multisubunit tether) couples vesicle arrival to immune SNARE function and how immune signaling dynamically tunes this relay [3], [5], [6], [24], [29].

(1) Snare-mediated vesicle trafficking during exocytosis

Plant exocytosis culminates in a four-helix SNARE bundle (Qa–Qb–Qc–R) that “zippers” opposing membranes together [9], [10], [34], [36]. Q-SNARE subfamilies are defined by helix position within the bundle: Qa (syntaxin-type) and Qb/Qc (SNAP-type) [36], [37]. During immunity, the PM Qa-SNAREs SYP121/penetration1 (PEN1) or SYP132 assemble with the Qbc-SNARE SNAP33 and vesicle R-SNAREs VAMP721/722 to deliver PR proteins (e.g., PR1), callose-associated machinery, and other antimicrobial cargo to pathogen attack sites [2], [20], [21], [22], [34].

This 3Q:1R *trans*-SNARE complex forms at PM nanodomains and can promote fusion even when only partially zippered, because SEC17/α-soluble NSF attachment protein (α-SNAP) and SEC18/*N*-ethylmaleimide–sensitive factor (NSF) can catalyze zipper completion and promote membrane merger, refining the classical “full-zipper” model [10], [38], [39], [40]. R-SNAREs of the VAMP7 (VAMP72) clade carry an N-terminal longin domain that can autoinhibit complex assembly; phosphorylation (e.g., VAMP721 Y57) and partner engagement likely relieve this constraint and license fusion at defense foci [38], [41].

Plants also encode expanded SNARE repertoires (approximately 55–60 genes in Arabidopsis versus about 20 in yeast), providing multiple Qa- and R-SNARE isoforms that can be deployed in parallel routes with overlapping but biased immune roles (e.g., SYP121- versus SYP132-centered pathways that converge on VAMP721/722-type vesicles) [8], [11], [38], [42], [43]. In polyploid crops, gene duplication further increases combinatorial capacity, supporting partially specialized secretory routes rather than a single universal “immune SNARE” module [8], [44], [45]. Together with genetic and in vivo interaction studies, these observations support a functional fusion hub in which exocyst-dependent tethering is coupled to SNARE-driven membrane fusion.

(2) The exocyst complex: composition, diversification, and immune roles

The exocyst is an octamer of SEC3, SEC5, SEC6, SEC8, SEC10, SEC15, EXO70 and EXO84 [46], [47]. In vivo, it can act as an octameric holocomplex or as two tetrameric subcomplexes that dynamically assemble at the cortex, creating a modular tethering platform [48], [49]. While yeast and mammals typically encode single copies, plants retain multiple paralogs (two each for SEC3, SEC5, SEC10, and SEC15; three for EXO84) and a markedly expanded EXO70 family (up to 47 EXO70 in rice) [47], [50], [51]. EXO70 is the most diversified subunit and is grouped into three sub-families (I-III) and eight major clades (A-H) [28], [29].

Sub-family I (mainly Clade A) preserves the canonical exocyst function: EXO70A binds phospholipids, recruits the exocyst to the PM, and links secretion with cell growth and cell wall biogenesis [29], [52]. Sub-family II shows extensive functional innovation (most clades B–F and H), including rapid lineage-specific diversification of clades F and H in monocots and dicots, respectively [28], [29]. In cereals, grass-specific EXO70F/EXO70FX members have acquired immune roles; notably, EXO70F/EXO70FX can partner with leucine-rich repeat receptor kinases (LRR-RKs) to mediate resistance [6]. This asymmetric expansion supports the idea that individual EXO70 isoforms can act as cargo- and context-specific tethers, while sub-family I maintains conserved roles in growth-associated secretion.

(3) How the exocyst stimulates tethering and primes SNARE-mediated fusion

The exocyst components SEC3 and EXO70 target phosphatidylinositol-4,5-bisphosphate (PI(4,5)P_2_)-rich PM nanodomains and tether incoming vesicles, increasing the local concentration of fusion-competent SNAREs [48], [52], [53], [54]. In vivo, EXO70A1 binds SYP121 and VAMP721; loss of EXO70A1 diminishes their PM residency, indicating that the tether helps position the fusion machinery [3]. Cortical actin and class XI myosins deliver vesicles to these nanodomains and prolong vesicle dwell time, increasing the probability of productive fusion [35]. Additional lipid cues (e.g., PI4P/sterol nanodomains) stabilize EXO70A1 and SYP121, reinforcing tether sites [3], [52]. Assembly of SEC5/SEC6–SEC8 subcomplex scaffolds can further strengthen the cortical capture platform and sustain carrier engagement at the membrane [55].

Immune signaling further modulates this relay. MPK3 phosphorylation of EXO70B2 promotes ATG8 binding and autophagic turnover, constraining fusion-competent domains after elicitation [24]. In parallel, Ca^2+^/synaptotagmin-5 (SYT5) can bias SYP132–VAMP721/722 assembly to increase throughput under immune demand [22]. These inputs tune both the efficiency and spatial precision of tethering and fusion at pathogen interfaces.

(4) Does the exocyst shape SNARE localization?

Certainly, in exo70a1 mutants, PM abundance of SYP121 and VAMP721/722 is reduced, consistent with a tether-positioning role for EXO70A1 [3], [56]. During infection, EXO70B2 and core exocyst subunits concentrate at papillae and encasements; disrupting EXO70B2 compromises these wall-based defenses and reduces focal recruitment of SYP121/PEN1–SNAP33–VAMP721/722 [17], [56]. Upstream, EXO70B1/B2-driven receptor delivery helps establish SNARE-permissive PM nanodomains, while the MPK3–EXO70B2–ATG8 axis can prune tether pools to reset SNARE residency as defense responses subside [24].

EXO70A1 can remain cortically localized even when SNAREs are perturbed, supporting a directional influence of the exocyst on SNARE positioning rather than the reverse [3], [51], [52], [57], [58]. Differential binding may also impose order: PM-resident SYP121 and vesicle-resident VAMP721/722 show distinct affinities for EXO70 isoforms, consistent with stepwise capture (VAMP–EXO70A1) followed by stabilization with Qa/Qbc partners at the cortex [3], [4], [18], [38], [59]. Likewise, perturbing EXO70A1 and EXO84 alters peripheral SYP121/VAMP721 residency and secretion outputs, linking exocyst integrity to SNARE distribution and recycling [4], [60], [61].

## Role of Rab GTPases

Rab GTPases are the largest branch of the Ras superfamily in plants and act as GTP-dependent molecular switches that choreograph secretory traffic from vesicle budding to fusion [62]. Upon activation by guanine-nucleotide exchange factors, Rabs recruit binding partners—including multisubunit tethers (the exocyst), cytoskeletal motors, and SNARE regulators—to specify when and where cargo is delivered [4]. During immunity this logic is re-deployed to rapidly redirect throughput to pathogen contact sites. Recent syntheses place the RabA (Rab11-like) and RabE (SEC4/Rab8-like) families at the heart of post-Golgi trafficking to the PM and emphasize their crosstalk with the exocyst and immune SNARE modules [4], [43], [63]. Functionally, this places RabA/RabE as timing modules that gate when tethering transitions to fusion.

(1) Rab–exocyst coordination in vesicle tethering

Across eukaryotes, SEC15 functions as a Rab11/8 adaptor within the exocyst, and plants follow the same architectural theme: RabA/RabE routes converge on exocyst subunits to stabilize vesicle tethering prior to SNARE engagement [63], [64], [65], [66]. Plant trafficking maps explicitly place Rab11/8 members upstream of SEC15 and holocomplex assembly at PM nanodomains, consistent with live-cell imaging at immune interfaces [63], [67]. This arrangement explains why Rab activation accelerates exocyst residency and increases vesicle capture kinetics.

(2) Rab–SNARE interplay in docking and fusion

Activated RabA2a directly recruits the VAMP721/722–SYP121–SNAP33 ternary complex, promoting assembly of the immune fusion machinery at the PM [4]. This shows that RabA2a can drive secretory fusion in parallel to—and in some contexts independently of—canonical exocyst pathways, providing a fast lane for defense secretion at attack sites.

(3) Regulation of Rab activity during immune signaling

During pathogen perception, Rab expression and localization are reprogrammed to favor defense cargo [68], [69]. Recent overviews report RabA/RabE up-regulation during PTI and document how oomycete effectors (e.g., *Phytophthora* RXLR24) bind plant RaBA proteins to subvert secretion [63], [70], [71]. Thus, Rabs are both drivers and targets in the plant–pathogen arms race.

(4) Rabs as integrators of cytoskeleton, tethering, and fusion

Rabs couple long-range transport to cortical tethering sites. In Arabidopsis, myosin XI–driven carriers interact dynamically with actin and the exocyst, forming a physical bridge from Rab-labeled vesicles to exocyst landing pads where SNAREs assemble [35], [38], [69]. This integration explains how secretion is focused to growth poles (e.g., root hairs, tip-growing cells) and to papillae/encasements during infection.

## Exocyst–SNAREs play key role in plant immune responses

Recent work underscores that secretion is not merely downstream of immune signaling; it is an execution module that determines whether defense cargo (PR proteins, callose machinery, antimicrobials) reaches pathogen contact sites in time [2], [72], [73]. Mechanistically, plants use a tether-handover-fusion sequence in which the exocyst concentrates post-Golgi vesicles at PM nanodomains and immune SNARE complexes drive focal fusion [3].

(1) Exocytic (TGN–PM) SNARE interactions during plant defense

Plant immune exocytosis at the PM is organized around Qa-SNAREs SYP121/PEN1 and SYP132, which assemble with Qbc-SNAREs such as SNAP33 (and SNAP32/SNAP34) and R-SNAREs VAMP721/722 [20], [22], [74], [75]. These complexes polarize to papillae and encasements at pathogen contact sites and are required for pre-invasive resistance—particularly against powdery mildews—by focusing secretion of antimicrobial metabolites and cell-wall reinforcements where the fungus attempts entry [17], [75], [76], [77].

Syntaxins usage can bias outputs by pathogen lifestyle. SYP121/PEN1 preferentially partners with VAMP721/722 during fungal attack, whereas SYP132 more prominently supports bacterial defense (see Fig. 2) [22], [77]. SYP132 is especially important for secretion of apoplastic immune cargo (PR1/PR2/PR5) and broader salicylic acid (SA)/jasmonic acid (JA)-responsive proteins during *Pseudomonas syringae* challenge; loss of SYP132 compromises this secretion, whereas overexpression enhances it [2], [22], [78]. In wheat, SYP132 can also pair with the TGN SNARE *Triticum aestivum* Novel Plant SNARE 11 (TaNPSN11) during stripe rust resistance, highlighting crop-level tuning of this pathway [79].

**Fig. 2:**
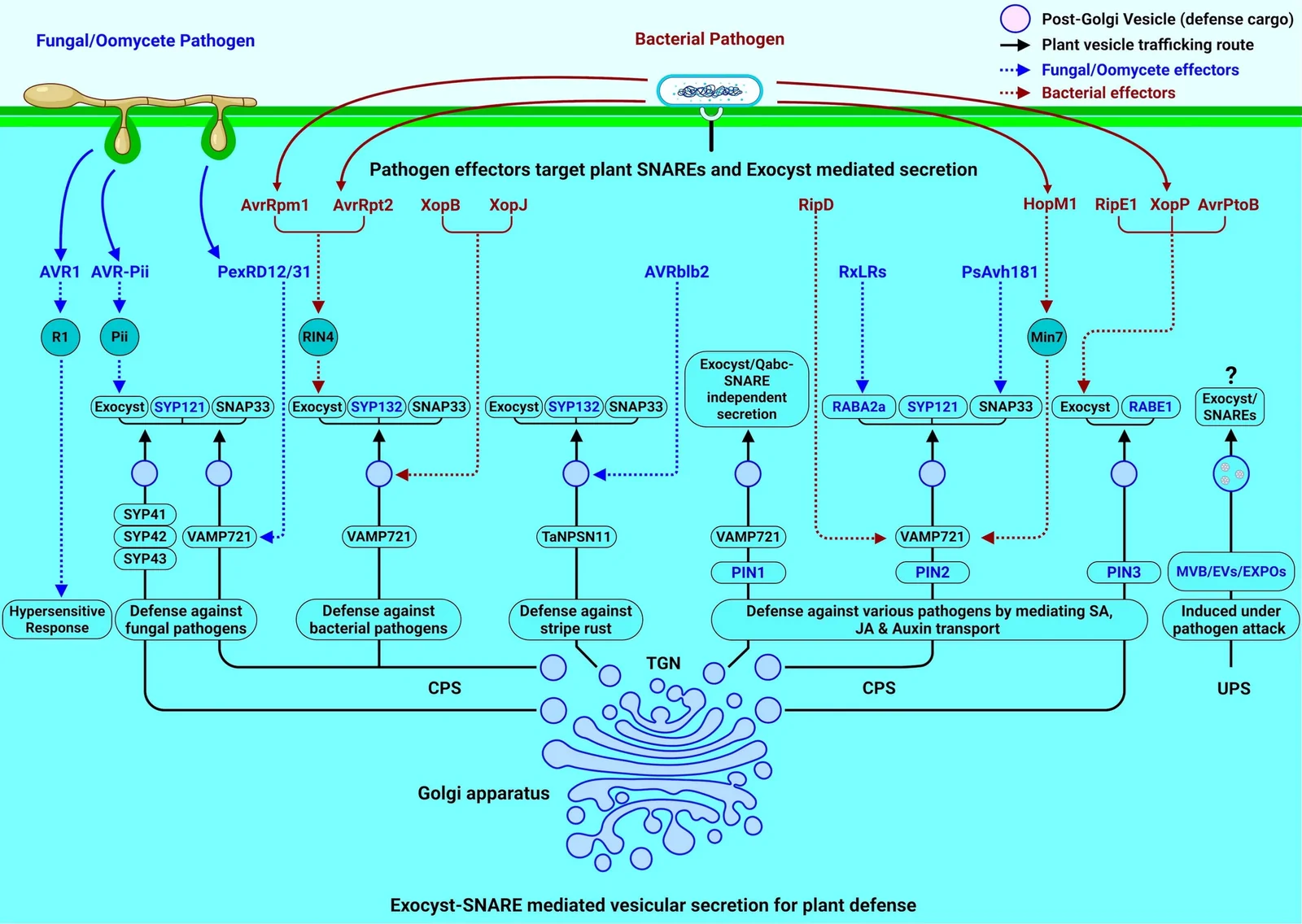
Pathogen effectors rewire exocyst–SNARE trafficking to shape plant defense across conventional protein secretion (CPS) and unconventional protein secretion (UPS) routes. This schematic summarizes how pathogens manipulate the plant secretory pathway from the TGN to the PM and how plants route immune cargo via exocyst–SNARE modules. Defense cargo is synthesized and sorted at the TGN and normally delivered by canonical/CPS to PM nanodomains where the ternary SNARE complex—SYP121/SYP132–SNAP33–VAMP721/722—mediates fusion. In some instances, upstream TGN Qa-SNAREs (e.g., SYP41/SP42/SYP43) may also take part in defense-related secretion, feeding post-Golgi vesicle traffic into exocyst–SNARE mediated fusion at infection sites. **Fungal/oomycete effectors (blue)** target this relay at multiple points: AVR1 binds the exocyst subunit SEC5 and suppresses PR1/callose secretion but is recognized by R1 to trigger a hypersensitive response; AVR-Pii binds EXO70F2/F3 and is sensed by Pii to confer resistance; PexRD12/31 interacts with VAMP72 to impede vesicle fusion; RXLR effectors can hijack RabA GTPases to divert post-Golgi traffic. **Bacterial effectors (red)** act on both receptor supply and the tether–dock–fuse steps: AvrRpm1 and AvrRpt2 modify or cleave Resistance to Pseudomonas syringae pv. maculicola 1 (RPM1)-interacting protein 4 (RIN4), perturbing exocyst/SNARE-linked signaling; XopJ and XopB broadly inhibit secretion; XopP prevents assembly of exocyst sub-complexes (SEC10/EXO70/EXO84), blocking tethering; AvrPtoB and RipE1 target EXO70B1 for degradation; HopM1 targets the ARF-GEF MIN7, reducing carrier formation upstream of the TGN; RipD binds VAMP721/722 to dampen PTI/ETI-associated fusion. The diagram also highlights route specialization: SYP121–SNAP33–VAMP721/722 focuses secretion against filamentous pathogens, whereas SYP132–SNAP33–VAMP721/722 (and the cereal TaNPSN11 module) bias PR and anti-bacterial cargo export. RabA2a can partner with SYP121–SNAP33 to deliver PIN2 and defense-related cargo in parallel to the exocyst, while RABE1 with the exocyst supports PIN3 trafficking; PIN1 can use a VAMP721-dependent route with limited exocyst/Q-SNARE involvement. Under strong challenge, plants also employ UPS via exocyst-positive organelles (EXPOs)/multivesicular bodies (MVBs)-derived extracellular vesicles (EVs), to supplement CPS; however, the role of exocyst-SNAREs in MVBs/EVs remains unknown. These interactions illustrate how pathogens cut the plant’s secretory “supply lines” and how plants switch among specialized CPS circuits and UPS to maintain focused delivery of PR proteins, callose machinery, and defensive metabolites at infection sites.

SYP121/PEN1 also contributes to effector-triggered immunity (ETI): *pen1* mutants can show reduced hypersensitive response (HR) to some bacterial and oomycete stimuli, implicating PEN1-dependent secretion in ETI [80]. PEN1 and its paralog SYP122 act partly redundantly—*pen1 syp122* double mutants show severe necrosis—yet they are not fully interchangeable, as SYP121/PEN1 more efficiently delivers papilla/encasement cargo [20], [76], [77], [81]. R-SNARE choice further refines routing: VAMP721/722 with PEN1 supports papilla formation, plasmodesmata-located protein 1 (PDLP1) delivery, and callose encasement at fungal entry sites [20], [41], [75], [77]. Upstream at the TGN, SYP4-family syntaxins (SYP41/42/43) help sort PRR and defense cargo into post-Golgi carriers that feed the PEN1/SYP132 routes, linking TGN export to immune secretion (see Fig. 2) [12], [82].

These modules indicate that the spatial organization and maintenance of SNARE nanodomains—supported by TGN sorting and exocyst-defined PM capture sites—underlie the micron-scale precision of antimicrobial secretion during infection. A key remaining question is how these SNARE nanodomains are organized and stabilized to enable such highly localized secretion.

(2) Role of exocyst subunits in plant defense

As outlined in Chapter 2, diversification of EXO70 isoforms provides a basis for context-specific tethering, and several exocyst components have clear immune functions. Loss of EXO70B2 compromises papillae and encasement formation and causes defects in both growth and immunity [16], [17]. In cereals, immune-active EXO70 variants such as EXO70FX12 demonstrate that exocyst specialization can be directly recruited into disease resistance [6].

Additional evidence supports exocyst involvement across pathosystems: rice EXO70F3 is guarded by the Pii–NLR pair [83]; actin–exocyst coupling via the myosin-XI motor OsMYA1 and OsEXO70H1 modulates blast immunity [5]; mildew resistance locus O 2 (MLO2) associates with EXO70B2 during mildew responses [84]; penetration 3 (PEN3) recycling to the PM requires EXO84 [85]; and EXO70E2 marks exocyst-positive organelles (EXPOs)/EV routes enriched for defense proteins [86], [87], linking the exocyst to unconventional secretion during infection. Regulatory feedback further constrains secretion: MPK3 phosphorylation of EXO70B2 increases ATG8 binding and autophagic turnover, limiting tether availability after elicitation and helping reset secretory capacity once demand subsides [24].

(3) Exocyst–SNARE convergence in plant immunity

At immune foci, tethering and fusion machineries converge to enable rapid, spatially restricted secretion. In Arabidopsis, EXO70A1 interacts with SYP121 and VAMP721 and helps position these components at the PM; loss of EXO70A1 reduces their cortical accumulation, supporting a tether-to-SNARE handover model [3], [18]. Consistent with this, *exo70a1* mutants mislocalize VAMP721/722 and SYP121 and show impaired SNARE-dependent secretion, while *exo70a1 vamp721* double mutants exhibit severe growth defects, highlighting an EXO70A1–VAMP721 functional unit important for growth and immunity [3], [18]. EXO70B1/B2 also cooperate with Qa-SNARE pathways to sustain PM levels of immune recognition proteins; reduced surface abundance in syp121 exo70b1 double mutants supports the idea that exocyst positioning helps organize immune complexes within PM nanodomains to enable fast, focused secretion [3], [16], [88]. Across crop pathosystems, this framework is broadly conserved but tuned by lineage, tissue context, and pathogen lifestyle.

### Exocyst–SNARE circuitry in hexaploid wheat

Polyploid cereals illustrate how genome duplication can expand—and then partition—exocyst–SNARE capacity into defense specialized and housekeeping routes. In Triticeae, including hexaploid wheat (Triticum aestivum, AABBDD), approximately 200 EXO70 genes have been catalogued [89], and wheat encodes approximately 173 SNARE genes, including expanded SYP1-class Qa-SNAREs and VAMP72-type R-SNAREs [8]. Expression profiling during powdery mildew infection indicates that only a subset of homeologs is strongly induced in resistant genotypes, consistent with subfunctionalization and defense biased deployment [8].

Direct molecular evidence further supports functional differentiation among wheat SNARE homologs. During stripe rust challenge, three closely related NPSN homologs (TaNPSN11/12/13) show differential induction, and virus-induced gene silencing (VIGS) demonstrated non-redundant contributions to immunity: knockdown of TaNPSN11 or TaNPSN13, but not TaNPSN12, reduced resistance to *Puccinia striiformis*. Importantly, interaction specificity was also demonstrated—TaSYP132 interacts specifically with TaNPSN11, but not TaNPSN12/13, in yeast two-hybrid (Y2H) and bimolecular florescence complementation (BiFC) assays [79]. This provides direct support for selective SNARE pairing and implies that only particular homologs are incorporated into defense-relevant trafficking routes rather than functioning as interchangeable copies. Consistent with this idea, during powdery mildew infection another defined SNARE pair (TaSYP137–TaVAMP723) shows physical interaction (Y2H) and functional divergence, and perturbation of this module alters host susceptibility [90], underscoring that duplicated SNAREs can specialize into distinct defense roles.

Comparable partitioning is evident for exocyst homologs in Triticeae. A genome-wide EXO70 analysis found that only a subset of wheat EXO70 genes is detectable expressed across tissues and/or responsive to biotic and abiotic triggers, consistent with regulatory diversification among expanded homologs [89]. Beyond expression, isoform-specific wiring is supported by interaction data: EXO70E1-V (from a wheat wild relative used in resistance genetics) physically interacts with the resistance-related factor CMPG1-V in multiple assays (pull-down, BiFC, LCI) and is inducible by powdery mildew and SA [91], illustrating selective recruitment of particular exocyst isoforms into defense signaling. Functionally, a lineage-specific EXO70FX12 isoform, together with its receptor-kinase partner Rps8/Pur1, promotes focused secretion toward haustorial entry sites during stripe rust infection, feeding into a SYP121–SNAP33–VAMP721/722 fusion module that reinforces the local cell wall [3], [6]. These findings support a “defense-biased subset” model in which inducible EXO70 and SNARE paralogs preferentially assemble immune secretion modules, while other retained copies primarily sustain essential housekeeping trafficking.

### Cotton: allotetraploidy and partitioning of exocyst–SNARE functions

Allotetraploid cotton (Gossypium hirsutum, AADD) similarly encodes an expanded exocyst–SNARE toolkit. Across four cotton species, 165 EXO70 genes have been identified and grouped into eight clades (EXO70A–H); 55 and 57 EXO70 genes in Gossypium barbadense and G. hirsutum, respectively [60]. Many paralogs exhibit strong tissue-biased expression, and silencing the PM-associated isoform GhEXO70A1-A alters thousands of transcripts—including defense-related pathways—supporting substantial functional divergence among retained copies [60]. At the fusion step, genome-wide analyses identified 51 GhR-SNARE genes; several GhVAMP72-type R-SNAREs are induced by abiotic stress and contribute to drought tolerance, illustrating how expanded SNARE sets can diversify to support both stress adaptation and immune-associated secretion [45]. In the *Verticillium* pathosystem, cotton recruits a conserved immune SNARE module (GhSYP121–GhSNAP33–GhVAMP721/722) in xylem vessels to promote resistance, consistent with portioned deployment of secretion machinery in vascular defense [14], [21], [45].

Molecular evidence further supports functional differentiation among SNARE homologs. GhSY121 is induced by *Verticillium dahliae* yet acts as a negative regulator of defense: loss-of-function enhances resistance, whereas overexpression increases susceptibility; moreover, GhSYP121 physically interacts with GhSNAP33, a cognate SNARE component required for penetration resistance [21]. These findings show that even within expanded SNARE families in an allotetraploid genome, individual homologs can acquire distinct regulatory roles and interaction specificities relevant to immunity. On the exocyst side, GhEXO70A1-A localizes to PM and shows selective interaction with specific EXO84 isoforms in Y2H assays [60], consistent with isoform-specific exocyst assembly and specialization in cotton.

### Rapeseed: exocyst–SNARE diversification in an allotetraploid oilseed crop

Rapeseed (Brassica napus, AACC) is an allotetraploid oilseed crop with one of the largest reported SNARE inventories. A genome-wide survey identified 237 SNARE genes spanning five canonical subfamilies (Qa, Qb, Qc, Qbc, and R) and 25 subclades [44]. Many BnaSNAREs display strong root- versus leaf-biased expression biased expression and are transcriptionally responsive to *Sclerotinia* sclerotiorum infection and immune-relevant treatments, including oxalic acid, SA, JA, and abscisic acid (ABA) [44]. RNA-seq and RT-qPCR further support differential regulation among SNARE homologs, including cultivar-dependent induction patterns during *Sclerotinia* challenge.

Co-expression analysis revealed coordinated induction of BnaSYP121/122, BnaSNAP members, and BnaVAMP722/723 isoforms during infection, and STRING-based interaction predictions are consistent with probable complex formation among these induced components—supporting the emergence of a defense-relevant SNARE module from polyploid expansion [44]. These expression and network-level data highlight BnaSYP121/122, BnaSNAP33, and multiple BnaVAMP72 isoforms as leading candidate immune SNAREs that likely cooperate with EXO70A1-like exocyst subunits to drive focal secretion in stems during *Sclerotinia* stem rot.

### Emerging principles from polyploid crops

Across wheat, cotton, and rapeseed, whole-genome duplication has expanded EXO70 and SNARE gene families, yet immune secretion typically draws on a restricted, inducible subset rather than all retained copies. Evidence for this includes divergent expression among homeologs, gene-specific immunity phenotypes, and selective protein–protein interactions (e.g., preferential SNARE–SNARE pairing or isoform-specific EXO70 associations) [44], [60], [79], [89]. Nevertheless, homeolog-resolved exocyst–SNARE interactomes remain largely unmapped, and systematic interaction profiling will be essential to establish causal, copy-specific wiring. Collectively, homeologs often partition by subgenome-bias, tissue specialization (e.g., epidermal versus vascular versus stem-cortex defense), and stress/pathogen responsiveness, creating a division of labor that enhances phenotypic flexibility and broadens targets for breeding or genome editing to strengthen secretion-based immunity with minimal fitness trade-offs.

Taken together, the host exocyst–SNARE module defined above functions as an immune “execution hub” that (i) concentrates secretory carriers at PM nanodomains, (ii) licenses SYP121/SYP132-dependent fusion with VAMP-type vesicles, and (iii) sustains focal delivery of wall-reinforcing and antimicrobial cargo at infection sites. By linking surface immune capacity with precisely focused secretion, these integrated host module functions also predict the most effective pathogen interference targets: effectors that destabilize EXO70 recruitment/holocomplex assembly, perturb SNARE pairing or its local microenvironment, or corrupt upstream trafficking regulators can simultaneously impair cargo delivery and dampen defense output, thereby weakening physical barriers and antimicrobial secretion. This functional coupling explains why exocyst-SNARE nodes repeatedly emerge as preferred targets—single, high-leverage choke points that coordinate where and when secretion occurs at the host–pathogen interface. In the next chapter, we map these host functions onto known effector strategies, highlighting how diverse pathogens converge on the same secretion node to suppress immunity.

## Pathogens decode plant immunity and disrupt supply lines

Pathogens do not merely evade recognition; they actively sabotage the secretory relay that executes defense. Recent work shows effectors can (i) block receptor provisioning by targeting EXO70 subunits, (ii) jam fusion hubs by interfering with immune SNARE routes or their assembly environment, and (iii) reroute traffic upstream by corrupting vesicle-trafficking regulators. The shared outcome is reduced PR1/PR2 export, weaker callose and lignin deposition at papillae, and a more permissive infection court.

(1) Choking receptor supply: Effectors targeting the exocyst

A landmark example is the *Xanthomonas campestris* core effector XopP, which binds plant EXO70B and blocks exocyst-dependent exocytosis without triggering the nucleotide-binding leucine-rich repeat receptor (NLR) that guards EXO70B in Arabidopsis [27], [93]. XopP suppresses PR1 secretion, callose deposition, and flagellin-sensing 2 (FLS2) delivery to the PM, effectively shutting down PTI at the tether step [27], [93]. In cereals, fungi also exploit EXO70: the rice blast effector AVR-Pii binds OsEXO70F2/F3; structural work on AVR-Pii–EXO70F2 complex revealed a distinct zinc-finger effector fold, underscoring convergent pressure on EXO70 as a host hub [92]. These interactions help explain the rapid diversification of EXO70FX in grasses and why immunity can hinge on single EXO70 alleles (e.g., barley EXO70FX12).

(2) Jamming fusion hubs: Hits on immune SNARE routes and their microenvironments

Powdery mildews deploys core effectors that target the PEN1/SYP121 pathway—a conserved defense route reliant on focal secretion at the PM. Recent plant-pathology updates detail how these effectors dampen pre-invasive immunity by disrupting endomembrane/PM trafficking needed to assemble SNARE microdomains [25], [26], [27], [92], [95], [96]. More broadly, contemporary reviews frame immune secretion as the execution arm of PTI/ETI, placing SNARE assembly, vesicle tethering, and receptor logistics directly in the line of fire for pathogen effectors [38], [46], [56], [97], [98]. This perspective rationalizes why exocyst–SNARE interfaces recur as effector targets.

(3) Rerouting the pipeline upstream: Trafficking regulators under attack

Bacterial effectors can rewire the pipeline before vesicles reach the tether. In P. syringae, HopM1 (with AvrE) promotes water-soaked lesions and suppresses defense by destabilizing the ADP-ribosylation factor (ARF)-guanine nucleotide exchange factor (GEF) MIN7, broadly impairing host secretion. Multiple syntheses reaffirm that the HopM1–MIN7 module is central to pathogen virulence, that small-molecule AvrE inhibitors attenuate disease in *hopM1* mutants, and that MIN7 is required to restrict *Fusarium* infection, underscoring the translational importance of trafficking control [99], [100], [101].

(4) Viral and nematode interference with exocyst–SNARE trafficking

Compared with bacteria, fungi and oomycetes, how viruses and plant-parasitic nematodes interface with the exocyst–SNARE module is less well defined, but available data already identify this machinery as a vulnerabe node [102], [103]. For viruses, direct mechanistic evidence shows that they can co-opt both exocyst-linked exocytosis and SNARE-mediated fusion during intercellular transport and replication. In tomato, tobamovirus movement protein (MP) binds RabE1a, which associates with the exocyst subunit SEC10b; RabE1a/SEC10b-dependent exocytosis promotes MP delivery, while AP2β–MP binding supports endocytic trafficking, and RabE1a disruption suppresses tobamovirus infection [102]. Tobacco Mosaic virus (TMV) further engages the fusion machinery through interaction of its 126 kDa replication protein with the Qa-SNAREs SYP22/SYP23, and depletion of SYP2-family members reduces TMV accumulation and cell-to-cell spread [104]; similarly, the Qc-SNARE SYP71 supports fusion of Turnip Mosaic Virus (TuMV)-induced replication vesicles with chloroplasts during organelle remodeling [105]. In parallel, plants can export small RNAs (sRNAs) and Argonaute (AGO)-associated silencing factors via EVs to suppress filamentous pathogens, raising the possibility that EV-mediated RNA trafficking contributes to to antiviral defense [106], [107]; however, direct in-planta evidence that AGO1–small interfering RNA (siRNA) complexes or other antiviral cargos use exocyst–SNARE-dependent or exosome-like routes remains limited.

Plant-parasitic cyst nematodes highlight the opposite side of this interface: they depend on an intact host vesicle cycle to establish and maintain metabolically active feeding sites [108], yet this dependence also creates vulnerabilities within SNARE cycling. In soybean cyst nematode (SCN) interactions, resistance at the Resistance to *Heterodera glycines* 1 (Rhg1) locus alters α-SNAP dosage and impairs α-SNAP–NSF function, disrupting SNARE-complex disassembly, vesicle recycling, and trafficking within syncytia and sharply reducing nematode fitness [39], [40], [109]. Beyond this host-genetic evidence, effector-level data indicate that cyst nematodes can directly engage SNARE-associated nodes: the SCN effector HgSLP-1 co-purifies with Rhg1 α-SNAP [110], and the beet cyst nematode t-SNARE–domain effector HsSNARE1 binds plant α-SNAP (AtSNAP2) and PR1, promoting disease [111]; notably, closely related HgSNARE1 variants can shift interaction outcomes and infection phenotypes [112]. Although the exocyst has not yet been directly visualized at feeding sites, sustained deposition of wall materials and nutrient transporters implies continuous tether–SNARE activity at the host PM, making exocyst–SNARE nodes plausibly convergence points for nematode effectors that remodel trafficking.

(5) Evolutionarily conserved secretion nodes as common targets across pathogen groups: Implications for plant immunity strategies

Diverse pathogens—including bacteria, fungi, oomycetes, viruses, and plant-parasitic nematodes—repeatedly target evolutionarily conserved secretion hubs that coordinate immune trafficking and shape defense output [36], [46], [47], [48], [50], [56]. These “pathogen-targeted secretion nodes” can be framed as: (i) cortical tethering (exocyst subunits and EXO70 isoforms that position vesicles at the PM and organize delivery at PM nanodomains), (ii) immune fusion hubs (PM SNARE routes centered on SYP121/SYP132–SNAP33–VAMP721/722 and their local assembly environment), and (iii) upstream trafficking logistics (Rab modules, ARF-GEF–dependent carrier formation, and TGN sorting) that determine whether immune cargos reach the cortex. This node-based view explains why unrelated pathogens often produce similar functional outcomes—reduced PR protein and cell-wall cargo delivery, weakened papilla/encasement reinforcement, and attenuated receptor-dependent immunity—by acting at different steps of the same relay.

Mechanistically, the evidence is strongest for bacteria and oomycetes, where multiple effectors directly target exocyst components (including EXO70 isoforms) or upstream regulators [25], [26], [27], [93], [113], [114], demonstrating that tethering and pathway initiation are exploitable bottlenecks; fungi likewise disrupt vesicle traffic and secretion-dependent wall reinforcement [92], even when the molecular resolution (direct binding versus indirect rewiring) is less complete. Notably, distinct pathogen groups often deploy different biochemical “weapons” to reach the same endpoint: bacterial effectors can inhibit or destabilize exocyst components at the tether step (e.g., XopP targeting EXO70, AvrPtoB-mediated degradation pathways) [27], [113], oomycete RXLR effectors directly engage exocyst subunits (e.g., SEC5 targeting) or broader trafficking modules identified by host-interactor screens [114], and fungal pathogens can impair vesicle fusion by acting on VAMP72-class R-SNAREs or SNARE-associated regulators [75].

Importantly, emerging work places viruses and nematodes within the same framework: viruses can engage Rab–exocyst-linked transport and SNARE-dependent fusion during movement, replication, and organelle remodeling, while cyst nematodes depend on host SNARE cycling and vesicle recycling to sustain feeding sites, with both host-genetic resistance (e.g., Rhg1-linked α-SNAP/NSF perturbation) and effector-level data indicating that SNARE-associated processes are manipulable hubs during parasitism [39], [102], [104], [105], [110], [111], [112]. These data support a shared principle: immune secretion hubs are high-centrality, high-payoff host interfaces under strong convergent selection by pathogens. Together, these patterns reveal a coherent pathogen playbook: cut the tether (EXO70/exocyst), muddy the fusion site (SYP121/SYP132-centered routes), and cripple upstream logistics (ARF-GEFs/Rabs/TGN sorting) —and, at specialized interfaces for viruses and nematodes, either exploit or rely on exocyst–SNARE-controlled secretion to sustain colonization.

This convergence motivates the core evolutionary question raised by this module: why are the same secretion hubs repeatedly targeted across kingdoms, and how can plants evolve resistance without compromising essential trafficking? Because exocyst- and SNARE-mediated secretion is ancient and indispensable, these nodes represent high-centrality constraints that pathogens can efficiently suppress with limited effector investment; conversely, plants appear to mitigate this vulnerability through isoform expansion and diversification (notably within EXO70 clades), pathway redundancy, and immune surveillance of vulnerable trafficking interfaces [6], [28], [29]. In rice, for instance, direct binding of a blast effector to a hydrophobic pocket in an EXO70 protein and its coupling to immune recognition illustrates how a conserved secretion node can become both an effector target and a surveillance point [92].

Practically, decoding these conserved targets defines actionable levers for durable resistance: (1) engineer or select effector-evading EXO70 alleles (e.g., editing EXO70B/FX interaction surfaces while retaining function), (2) reinforce SNARE fusion capacity (stabilizing SYP121/SYP132 routes under stress to sustain immune secretion), and (3) harden upstream trafficking regulators (e.g., MIN7/ARF-GEF modules via resilient alleles or guard-enabled surveillance). More broadly, this framework predicts that durable resistance can arise from (i) allele-specific remodeling of effector-binding surfaces on key nodes, (ii) reinforcement or redundancy in fusion routes, and (iii) immune surveillance (“guard/decoy”) of vulnerable trafficking interfaces. Integrating viral and nematode systems into this framework further suggests that exocyst–SNARE hubs and their regulators should be evaluated not only for resistance to bacteria, fungi and oomycetes, but also for their potential to limit virus spread and reduce nematode feeding success, thereby extending the translational value of secretion-node engineering across pathosystems.

## Knowledge gaps

Despite rapid progress, the handover from exocyst-mediated tethering to SNARE-driven fusion during immune secretion remains poorly defined. Fundamental questions still lack answers, such as how many vesicles are are tethered per fusion event, what their dwell times are, and whether Qa- and R-SNAREs assemble sequentially or in parallel at immune foci. In Arabidopsis, EXO70A1 associates and co-localizes with SYP121 and VAMP721/722 at pathogen contact zones, supporting a model in which the exocyst docks TGN-derived vesicles and organizes a fusion-competent SNARE module at secretion hotspots, with SYP121 potentially providing a regulatory surface [3], [28], [122], [123]. However, it remains unresolved whether EXO70A1 binding promotes the conformational transitions required for full SNARE zippering in vivo, whether the Qa domain of SYP121 is the decisive fusion interface, and how these events are temporally ordered; imaging further suggests that partially assembled exocyst subcomplexes can engage SNARE components, arguing against a strict “complete exocyst, then SNAREs” sequence and instead favoring parallel progression toward fusion competence [3], [124], [125], [126].

A second major gap is how stress-induced TGN sorting channels immune cargos—PR proteins (e.g., PR1), callose synthases and other antimicrobial factors—into SYP121- versus SYP132-centered secretion routes or toward EV-associated export, because a unified routing logic is still missing. PR1 clearly enters the secretory pathway, and its C terminus supports secretion and CAPE peptide release [73], [127], yet no determinant has been identified that assigns PR1 specifically to one Qa-SNARE route. Current evidence instead points to several candidate sorting layers. PR1 is delivered through the ER–Golgi–TGN pathway via its N-terminal signal peptide, and luminal maturation events (e.g., disulfide formation and N-glycosylation) can influence folding, quality control and secretion competence [73], [128]. At the TGN, trafficking is organized into subdomains (“zones”) that are segregated by adaptor machinery (e.g., adaptor protein complex 1 (AP-1)/clathrin secretory zones versus AP-4 vacuolar zones): membrane cargos can be selected via canonical cytosolic motifs (tyrosine-based, dileucine-type), whereas soluble cargos likely depend on luminal features and/or poorly defined capture mechanisms [129], [130], [131]. In parallel, the sterol/lipid-binding capacity of PR1 CAP domains suggests that protein–lipid interactions may bias partitioning into particular TGN/PM microenvironments and potentially EV-favored routes [132], [133], while EV/MVB-linked export is further shaped by endosomal sorting complex required for transport (ESCRT)-mediated sorting and membrane composition [134], [135], [136].

Consistent with this view, pathway choice is often framed as an emergent property of vesicle identity and membrane context: Rab GTPases and EXO70 isoforms are proposed to recruit vesicles to distinct PM sites, where they engage specific SNARE complexes [3], [4], [52]. However, decisive in-planta evidence for cargo-selective receptors/adaptors—or for direct cargo competition for EXO70 that would enforce Qa-SNARE route selection—remains limited. These mechanistic uncertainties extend upstream into the diversification of trafficking components. Although expression and phenotype studies document extensive EXO70 expansion, the molecular determinants that confer functional specialization (including lipid interactions and binding interfaces) are still poorly defined, and the specific Rab–exocyst–SNARE combinations that execute immune secretion remain largely unmapped. This becomes especially consequential in polyploid crops such as wheat, cotton, and rapeseed, where both EXO70 and SNARE families are further expanded but homeolog-resolved wiring maps are missing. As a result, we do not know which copies assemble into functional circuits in particular tissues and pathosystems, or how duplication has partitioned essential secretion versus immune deployment—information required to engineer resistance without compromising core trafficking.

Comparable gaps constrain our understanding of EV-mediated defense secretion. It is unclear whether the exocyst acts during cargo loading at the TGN, during tethering of EV membranes at the PM, or at multiple steps. Likewise, although PM SNAREs clearly drive conventional fusion, their direct involvement in EV release from MVBs or other unconventional carriers remains unproven, leaving the molecular basis of the transition from classical secretion to EV-associated export during sustained immunity undefined. More broadly, the relationship between conventional exocytosis and EV pathways—whether they function independently, intersect, or share modules only under specific conditions—remains unresolved. Most mechanistic insight also comes from leaf epidermis under controlled conditions, highlighting the need for tissue- and wall-context tests (roots, vasculature, lignified cells) and field validation to evaluate costs and benefits in realistic environments.

Pathogen interference with these pathways remains underexplored. While several effectors target EXO70 or upstream regulators such as ARF-GEFs, we still lack a coherent, cross-pathosystem map of how diverse pathogens (bacteria, fungi, oomycetes, viruses and plant-parasitic nematodes) converge on shared trafficking hubs and host interfaces. A key need is therefore a comparative framework for secretion-node targeting that distinguishes truly “core” exocyst/SNARE interfaces (recurrently targeted across kingdoms) from lineage-, tissue-, or context-specific vulnerabilities. Although direct virus- and nematode-linked interactions with trafficking components have now been documented in a few systems [39], [40], [102], [104], [105], [110], [111], [112], it remains unclear how broadly—and by what logic—viral proteins and nematode effectors converge on specific exocyst isoforms and immune SNARE routes in planta. Key questions include whether antiviral silencing complexes (e.g., AGO1–siRNA) are packaged into EVs via exocyst-dependent routes, whether exocyst subunits or immune SNAREs operate at viral replication sites, plasmodesmata, or nematode feeding structures; and which host trafficking factors are targeted to sustain vesicle recycling at syncytia [39], [40], [103], [106], [108], [109]. Addressing these gaps will require live imaging at viral and nematode interfaces, targeted loss- and gain-of-function perturbations of EXO70 isoforms, core exocyst subunits, and immune SNAREs, and systematic effector–host interaction screens mapped onto exocyst/SNARE/Rab networks. These datasets should be complemented by evolutionary analyses (e.g., clade- and residue-level election mapping) to pinpoint interface regions that diversify under pathogen pressure while preserving essential trafficking.

Closing these gaps will require approaches that explicitly connect molecular assembly to secretion dynamics at immune foci. Importantly, proof-of principle studies already demonstrate the power of this strategy: near-PM live imaging in Arabidopsis revealed exocyst subunits concentrated in discrete cortical foci with rapid turnover, consistent with transient tethering “hotspots” [18], [52], and quantitative high-speed imaging of endogenously tagged exocyst components in mammalian cells supports a dynamic, stepwise assembly model with subcomplex recruitment and departure occurring on distinct timescales at fusion sites [124]. Building on this foundation, future work should combine dual-color single-particle tracking with split-proximity labeling at immune foci to link exocyst/SNARE dwell times, assembly order, and fusion outputs with their local proteomic neighborhoods (e.g., TurboID/miniTurbo and split-TurboID toolkits) [137], [138], [139]. These efforts should be integrated with CRISPR-based domain swaps and allele series across EXO70–SNARE interfaces; multiplex mapping of Rab/SEC15/SNARE interaction networks; time-resolved phosphoproteomics aligned with quantitative residency and fusion metrics. In parallel, genetic dissection of EV biogenesis in trafficking mutants, cross-pathogen effector screens, and multi-environment trials that link secretory capacity to agronomic performance will be essential to translate mechanistic insights into durable resistance. For polyploid crops, these strategies must be extended to homeolog–resolved analyses to identify which exocyst–SNARE combinations carry immune load versus essential secretion, and for viral and nematode systems, to pinpoint trafficking nodes that can be rewired to restrict pathogen success without compromising vital cellular export.

## Future prospects and conclusions

Over the past decade, work across multiple systems has established the exocyst and SNAREs as a coordinated secretion module that underpins the speed and spatial precision of plant immune responses. By focusing vesicle delivery at pathogen contact sites and catalyzing membrane fusion, this partnership enables timely deployment of defense outputs, including PR proteins, callose-associated machinery, and antimicrobial metabolites. The next step is to move beyond static associations toward a mechanistic, real-time view of how tethering transitions into fusion in living cells, and how immune signaling tunes this relay to maximize defense while limiting growth penalties.

Meeting this challenge will require experimental pipelines that directly connect molecular interactions to dynamic secretion behavior. High-resolution live imaging—paired with CRISPR-based endogenous tagging—can track vesicle progression and protein recruitment at immune sites with minimal perturbation. In parallel, proximity labeling has moved from “promising” to “practical.” TurboID/miniTurbo has been validated in plants to recover protein neighborhoods and organellar proteomes in vivo, including from rare cell types, enabling capture of transient or weak associations that are difficult to preserve biochemically [137], [138]. Split-TurboID variants further enable contact- or event-dependent labeling, providing a direct strategy to map exocyst/SNARE-proximal regulatory layers (e.g., Rab adaptors, kinases, lipid-binding factors) specifically at immune secretion sites [139]. Together with time-resolved phosphoproteomics, these approaches should clarify the regulatory logic that gates secretion— spanning post-translational control, lipid-dependent cues, and small-GTPase signaling—and yield a causal framework linking immune signaling inputs to changes in exocyst–SNARE engagement, vesicle throughput, and defense output during pathogen attack.

Translational opportunities are equally clear. Rather than constitutively amplifying secretion, the field can focus on context-aware engineering strategies that strengthen defense with minimal chronic cost, such as selecting or redesigning EXO70 and immune-SNARE alleles to reduce effector sensitivity, tuning expression to stress-responsive windows, and editing regulatory sites that sharpen activation dynamics. In polyploid crops, expanded gene repertoires provide additional flexibility: distinct homeologs and paralogs can be prioritized for specific tissues and disease pressures, enabling tailored immune secretion modules while preserving essential trafficking. Designing and testing these alternative circuit configurations represents a tractable path toward durable, low-penalty resistance.

Ultimately, success will depend on multi-scale validation. Quantitative cell biology must be linked to performance across environments through greenhouse and field trials that capture variation in climate and microbiomes, supported by physiological modeling to balance defense gains against fitness trade-offs. In sum, the exocyst–SNARE partnership is both a core organizing principle for immune secretion and a practical entry point for crop improvement; integrating mechanism with deployment can turn this molecule into a tunable platform for resilience in a rapidly shifting pathogen landscape.

## Funding source

This work is supported by Key Program of Natural Science Foundation of Jiangxi Province (S20258731); 10.13039/501100004479Jiangxi Natural Science Foundation (20224BA205006); and Talent’s program of Jiangxi Province (jxsq2023101036).

## AI-assistance disclosure

Artificial intelligence tools (e.g., ChatGPT) were used under author supervision to improve language and structure; all scientific content and interpretations were checked and verified by the authors.

## Compliance with Ethics Requirements

This review does not report new experiments involving human participants, animals, or field sampling; therefore, institutional ethics approval and informed consent were not required. All sources are properly cited. The authors adhere to *Journal of Advanced Research* policies on authorship, originality, data integrity, and competing interests; disclosures are provided in the manuscript. AI assistance (ChatGPT, OpenAI) was used only for language polishing; no scientific content or data were generated or copied, and the authors take full responsibility for the manuscript’s accuracy.

## CRediT authorship contribution statement

**Muhammad Adnan:** Conceptualization, Writing – original draft, Software. **Liu Xiaokun:** Conceptualization, Supervision, Funding acquisition, Project administration, Writing – review & editing.

## Declaration of competing interest

The authors declare that they have no known competing financial interests or personal relationships that could have appeared to influence the work reported in this paper.
